# A Simple Power Law Governs Many Sensory Amplifications and Multisensory Enhancements

**DOI:** 10.1038/s41598-018-25973-w

**Published:** 2018-05-16

**Authors:** Vincent A. Billock, Paul R. Havig

**Affiliations:** 1 College of Optometry, Ohio State University, Columbus, OH 43210 USA; 2 U.S. Air Force Research Laboratory, Wright-Patterson Air Force Base, OH 45433 USA

## Abstract

When one sensory response occurs in the presence of a different sensory stimulation, the sensory response is often amplified. The variety of sensory enhancement data tends to obscure the underlying rules, but it has long been clear that weak signals are usually amplified more than strong ones (the Principle of Inverse Effectiveness). Here we show that for many kinds of sensory amplification, the underlying law is simple and elegant: the amplified response is a power law of the unamplified response, with a compressive exponent that amplifies weak signals more than strong. For both psychophysics and cortical electrophysiology, for both humans and animals, and for both sensory integration and enhancement within a sense, gated power law amplification (amplification of one sense triggered by the presence of a different sensory signal) is often sufficient to explain sensory enhancement.

## Introduction

Sensory enhancement or amplification is common when one sensory activity occurs in the presence of another^1–24^. For example, lights can appear brighter when a sound comes from the same direction in space^4^. Often that amplification seems to be intensity-dependent, with weak stimuli being amplified more than stronger stimuli. Moreover, neural systems responding weakly to a single stimulus often have more enhanced multisensory neural responses than neurons with strong unisensory responses. In sensory integration this is called the Principle of Inverse Effectiveness^3^, but this can also be true for amplification that occurs within a sense. Amplification is studied in many ways and presented in many formats, but has thus far evaded lawful quantification. Here we find a curious near-universal invariance: the amplified response is a power law function of the unamplified response, with a compressive exponent that explains much of intensity-dependent enhancement. The only exception we have found is for bimodal cells, especially in superior colliculus. For both psychophysics and cortical spike rate data, power law exponents are clustered around 0.85. This is true for vision amplified by auditory modulation and for audition amplified by somatosensory information. This is also true for amplification seen within a sense (in this case for human color vision). Similar power laws govern other electrophysiological measures. The power laws’ r^2^ values are generally extremely high, suggesting that gated (triggered) amplification, not nonlinear combination, is often sufficient to explain sensory enhancement.

## Results

**Figure 1:**
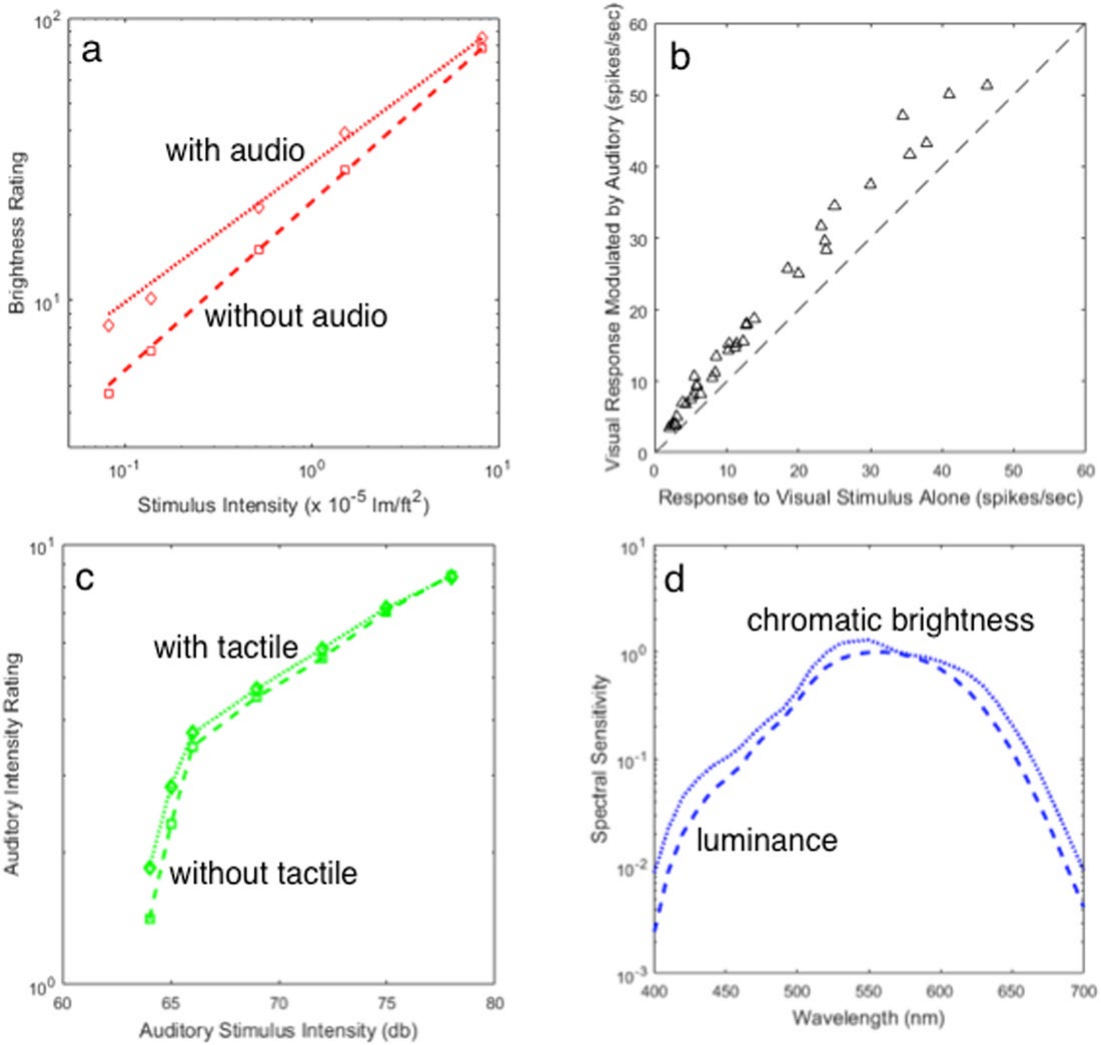
Data in various formats from four studies of intensity (or sensitivity) dependent sensory amplification. Fascinatingly, all four datasets turn out to obey the same law (Eq. 1, see Fig. 2). (**a**) Ratings of the brightness of five light intensities are higher when a sound comes from the direction of the light^4^. This is the single most compelling dataset on perceptual enhancement – note how the variable gap between the enhanced and unenhanced functions beautifully illustrates the Principle of Inverse Effectiveness (which is not always as clear-cut as found here). (**b**) Sub-threshold multisensory neurons in cat visual cortex (area PLLS) fire for visual stimuli, do not fire for audio, but have higher firing rates when both stimuli are present^17^. (Dashed line shows the unamplified baseline). (**c**) Subject ratings for loudness of seven audio signals are higher if the subject’s hand is also stimulated^10^. (**d**) Colored lights at short and long wavelengths look brighter^32^ (dotted function) than would be predicted based on their luminance^33^ (dashed function) − a performance-based measure. This spectral broadening (enhancement) of chromatic brightness spectral sensitivity relative to luminance spectral sensitivity has been thought to be due to a neural weighted combination of hue and luminance responses, but an alternate explanation can be seen in Fig. 2.

**Figure 2:**
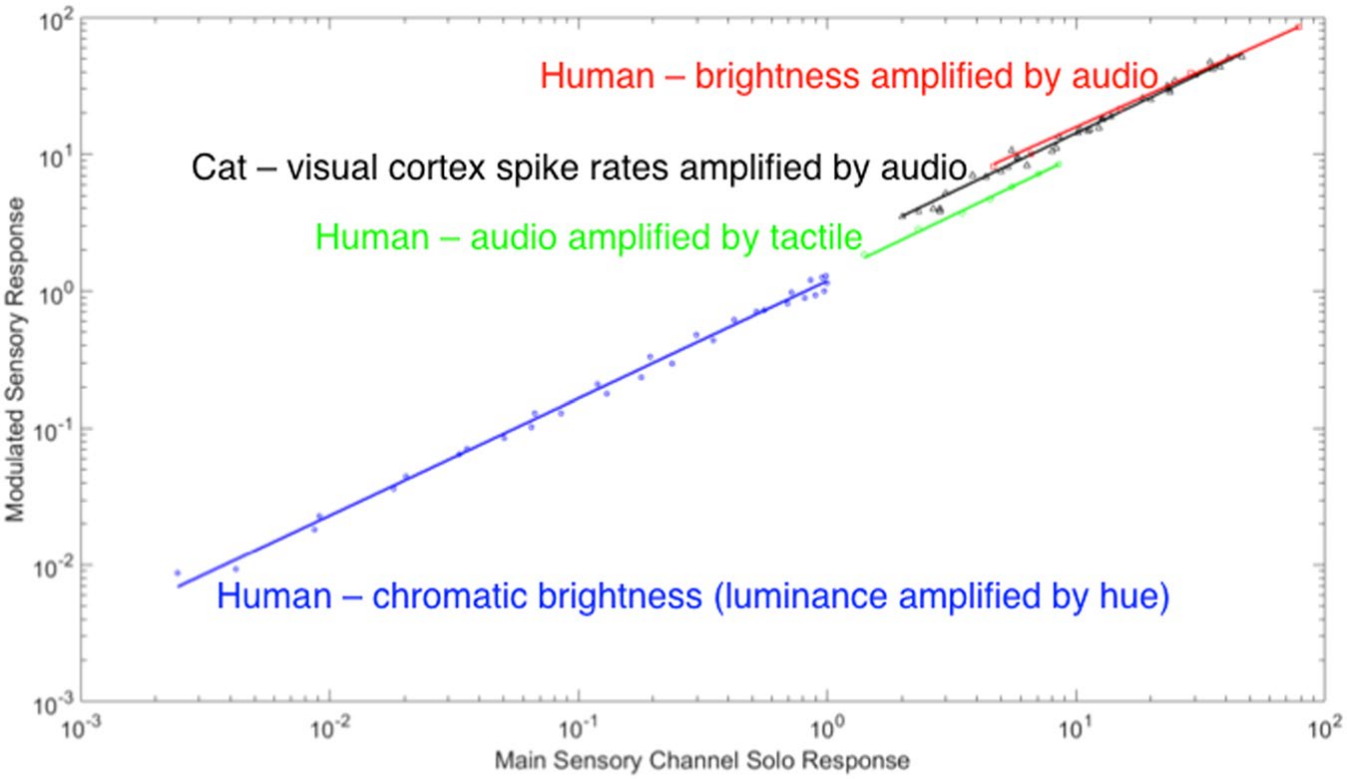
Amplified sensory responses are power laws of unenhanced responses. The four data sets shown here are the same color coded data shown in Fig. 1, modeled by Eq. 1. The slope of these power laws varies but slightly (Table 1). Note on units: The neural data are in spikes/sec for both axes; the psychophysical data scales are arbitrarily set by the original experiments (see Fig. 1). The fits have no units - ‘a’ and ‘n’ are dimensionless. The blue data are computed by plotting chromatic brightness spectral sensitivity as a function of luminance spectral sensitivity, using their common wavelengths to index the data.

Data showing that sensory signals are often enhanced or amplified in the presence of another sensory activity are shown in Fig. 1. The circumstances and nature of the amplification are diverse, but weakly effective stimuli generally receive relatively more amplification – the influential Principle of Inverse Effectiveness from sensory integration^3^. The details of inverse effectiveness are a subject of some controversy in sensory integration^11,18,20^, but it is a useful rule-of-thumb and the nature of intensity-dependent amplification is a matter of importance; a more general and fundamental rule for amplification would be welcome. Such a rule is shown in Fig. 2, where all four data sets from Fig. 1 are plotted in the same framework. Here the amplified or enhanced response is plotted as a function of the unamplified response. All four amplification functions − despite their differences in Fig. 1 − turn out to be power laws of the form,

1 $$Enhanced\,Response=a\ast Unenhanced\,Respons{e}^{n}.$$

Note that because both amplified and unamplified response have the same units, the fitting parameters *a* and *n* are unitless. This may be useful for comparing electrophysiology and psychophysics.

### Implications of all four cases following the same rule

There are four immediate implications of these findings. First, it is unlikely that the amplification effects stem from attention, which is also known to enhance sensory signal processing^25^. Compare (in Fig. 2) visual brightness amplification via auditory modulation (the red squares and line) to the firing rate increase in cat visual neurons (black triangles and line) when an auditory signal is present. Because the neural data was collected in anesthetized cats^17^, attention (in the classic sense) is unlikely to be involved. (We acknowledge the existence of preconscious elements to the range of phenomena involved in attention, but doubt their applicability here). By extension, the similarity of the psychophysical brightness amplification function to the neural data suggests that whatever neural mechanism amplifies the neural response in unconscious cats would suffice to account for the human psychophysical data as well.

Second, the quality of the fits in Fig. 2 are superb, with r^2^ values that range from 0.989 to 0.999 (Table 1). Since almost all of the variance is accounted for by a function of a single variable, the enhancements seen here are consistent with simple gated amplifications (*a*) of the unenhanced responses. The role of the modulating stimulus is to trigger the amplification of the modulated stimulus. It has long been known that there are essentially two classes of multisensory neurons: true multimodal neurons (bimodal and trimodal) and subthreshold multimodal neurons (also known as modulated unisensory neurons). True multimodal neurons, prominent in midbrain (superior colliculus/optic tectum) but also found in cortex, produce amplifications based on two or more inputs, and can produce remarkable amplifications (as much as 1200%^3^). The subthreshold multimodal neurons fire to only one kind of sensory stimulus, but are modulated by another, are studied mainly in cortex, and produce more modest amplifications, roughly a factor of two for weak stimuli in many studies^15,17,19^. It has been argued (based on refs^6,7,17^) that the modest amplifications produced by subthreshold neurons are more in keeping with amplifications seen in psychophysics than the remarkable amplifications produced by true multimodal neurons^24^. The data in Fig. 2 are consistent with that argument. The neural data shown in black in Figs 1 and 2 are from subthreshold multisensory neurons and the form of the subthreshold neurons’ amplification function is quite similar to the most relevant psychophysical data (red).

**Table 1:** Power laws (Eq. 1) for every analyzed sensory, cognitive and neural data set.

Conditions/[Reference #]	*a*	*n*	r^2^
**Psychophysics (rating scales & matching)**			
Visual brightness modulated by audio stimulation^a ^^4^	2.407	0.818	0.999
Audio loudness modulated by tactile stimulation^a ^^10^	1.300	0.873	0.998
Brightness of luminance modulated by hue (2°)^a,c ^^32^	1.198	0.864	0.993
Brightness of luminance modulated by hue (10°)^c ^^32^	1.282	0.833	0.993
*Average slope: psychophysics*		*0.847*	
**Subthreshold visual cortical neurons: cat PLLS (spikes/sec)** ^**d**^			
vision modulated by audio (55 db)^a ^^17^	1.939	0.868	0.989
vision modulated by audio (66 db)^15^	1.605	0.913	0.934
vision modulated by audio (75 db)^15^	2.175	0.837	0.911
vision modulated by audio (81 db)^15^	2.004	0.863	0.925
vision modulated by audio (55 db)^9^	2.212	0.822	0.986
*Average slope: spiking subthreshold neurons*		*0.861*	
**Cat bimodal neurons: (spikes/sec**)^**b,d** ^^23^			
Superior colliculus: best sense modulated by other^b ^^23^	4.524	0.654	0.628
Cortex – AES: best sense modulated by other^b ^^23^	2.111	0.843	0.830
Cortex – AES: best sense modulated by other^19^	3.427	0.687	0.864
Cortex – PLLS: vision modulated by audio^9^	2.114	0.831	0.911
Ave slope: cortical bimodal cells		0.754	
***Macaque auditory cortex, modulated by vision*** ^**d,e** ^ ^16^	2.536	0.773	0.690
**Macaque auditory cortical area A1 (audio modulated by tactile electric stimulation)** ^12^			
Supergranular layer, Current Source Density^b^	1.676	0.590	0.992
Granular layer, Current Source Density	1.126	0.669	0.989
Infragranular layer, Current Source Density	1.040	0.797	0.973
Supergranular layer, Multi Unit Activity^b^	1.352	0.646	0.980
Granular layer, Multi Unit Activity	1.951	0.597	0.981
Infragranular layer, Multi Unit Activity	1.034	0.786	0.998

^a^Also plotted in Figs 1 and 2.

^b^Also plotted in Fig. 3.

^c^Luminance is energy-based physiologically-motivated spectral sensitivity^33^ from www.cvrl.org and brightness is from Table 1 of [ref.^32^]. Both datasets measured for 2° and 10° visual fields.

^d^All spike rate data is converted from spikes/trial to spikes/second.

^e^Ref.^16^ did not characterize their neurons as subthreshold or bimodal; based on a few highly superadditive cells, there is likely a mixture of both types.

Third, a psychophysical measure − chromatic brightness spectral sensitivity − can be predicted from luminance spectral sensitivity; chromatic brightness is well described by a simple power law of luminance. Moreover this amplification function that describes chromatic brightness spectral sensitivity as a simple amplification of luminance spectral sensitivity (Fig. 2, blue data) follows the same rule as sensory integration data, suggesting similar (perhaps generic) amplification mechanisms can mediate both multisensory integration and sub-modal interactions (in this case hue-luminance interactions) within a single sense like color vision. This surprising result requires some background to be properly appreciated. Luminance spectral sensitivity and chromatic brightness spectral sensitivity were originally alternative ways of probing the spectral effectiveness of different color lights. Luminance was measured by using criteria that detected spatial or temporal discontinuities under conditions that discount color vision; chromatic brightness was measured by equating apparent brightness of different wavelength stimuli^26^. Luminance turned out to predict human performance (e.g., acuity) better, while chromatic brightness was the better predictor of color appearance. Chromatic brightness as a function of wavelength is broader than luminance; blue and red lights are brighter than they are luminous^26^. Luminance spectral sensitivity is associated with the spectral sensitivity of the achromatic channel (and can be measured physiologically), but there is no known color vision mechanism that corresponds to chromatic brightness. Because hue-opponent mechanisms like the red-green and blue-yellow channels have strong responses in the long- and short-wavelength (reddish and bluish) ends of the spectrum where luminance is weak, it has long been thought that chromatic brightness might be a weighted combination of hue and luminance responses and there is evidence that hue and luminance signals combine synergistically (via probability summation) at threshold. In particular, vector models of chromatic brightness have been popular (see refs^26,27^ for review). In these models chromatic brightness is the vector sum of weighted contributions of luminance and the absolute magnitude of the hue opponent (red-green and blue-yellow) channels. However, the fit of the power law amplification function in Fig. 2 contradicts theories that suprathreshold chromatic brightness is a combination of luminance and hue signals. Chromatic brightness is almost completely accounted for as a simple power law amplification of luminance (r^2^ = 0.993) leaving little variance for the hue channels to account for. The fit suggests that chromatic brightness is a simple amplification of luminance; likely this amplification is triggered by the presence of the hue signal, just as the visual neurons in Fig. 1b fire harder for vision stimuli when auditory stimuli are present.

**Figure 3:**
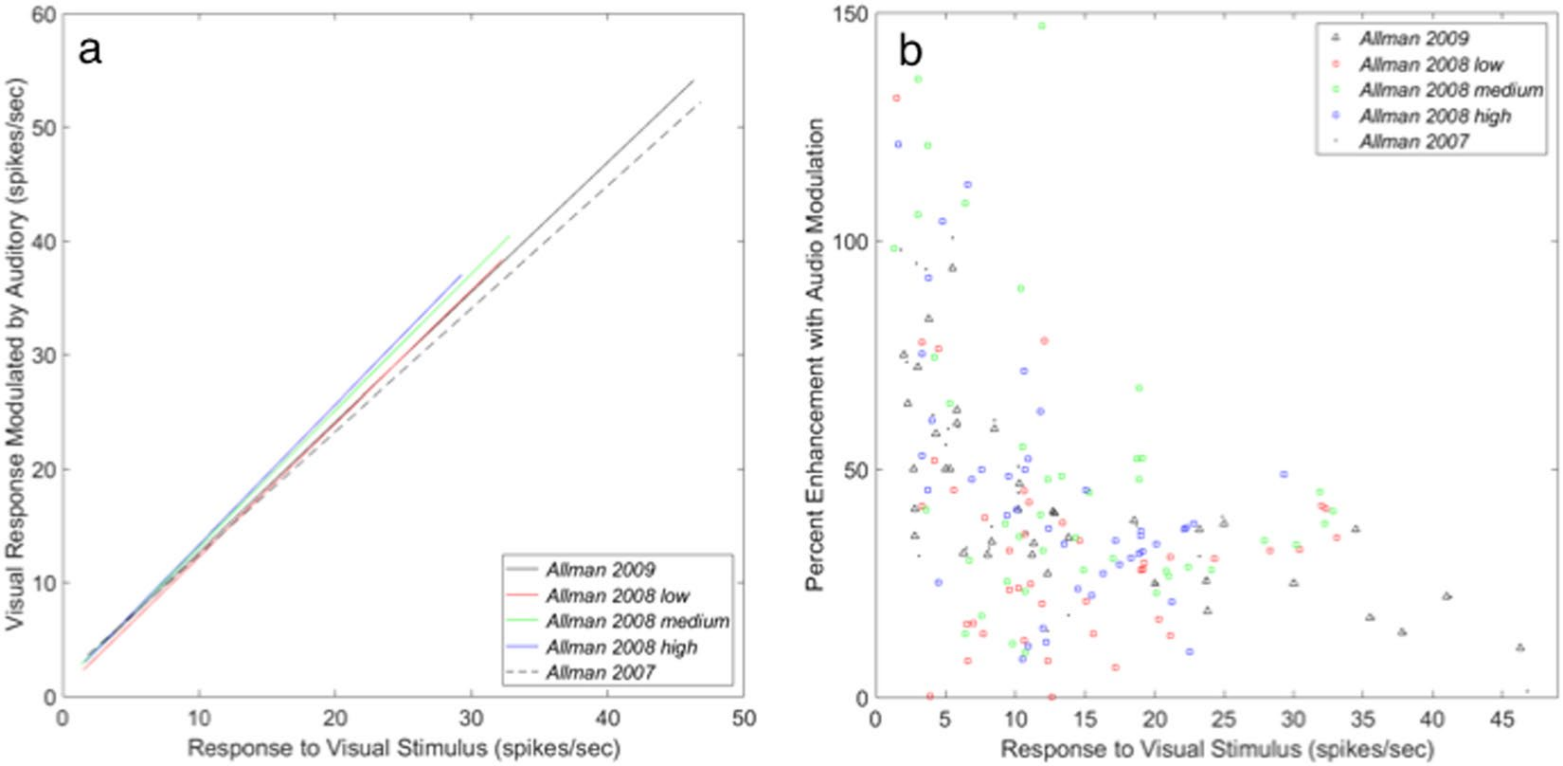
Repeatability and inverse effectiveness. (**a**) Repeatability - power law fits to similar cells from the same cortical area. Shown are power law fits to five neural data sets taken from three studies, each in cat visual cortical area PLLS^9,15,17^. The three datasets from ref.^15^ use the same neurons for different kinds of modulation. All neurons are subthreshold multisensory cells (see Table 1 for parameters). The dataset from Fig. 1b is the solid black line here. (**b**) Same data plotted as percent enhancement to illustrate the Principle of Inverse Effectiveness. Although there is neural variability, there is a strong tendency for cells with lower firing rates to have more firing rate enhancement when an auditory signal is presented with the visual stimulus. The dataset from Fig. 1b is shown as black triangles.

Fourth, the mildly compressive exponent *n* shown by all four data sets results in stronger amplification for weak (or weakly effective) signals than for strong ones. This is broadly consistent with the Principle of Inverse Effectiveness, but does not clarify some details like the extra enhancement of some weak − but not weakest − stimuli found in some studies^11,13,17,18,20^. Some examples of this can be seen in in Fig. 1b and in Fig. 3, which features data from cat extrastriate visual cortex. These are modulated unisensory neurons that respond to visual stimuli, do not respond to auditory stimuli alone but show enhanced spike rates when both visual and auditory stimuli are present. If we replot the data in Fig. 3a as percent enhancement in Fig. 3b, there is much neural variability, but it can be clearly seen that most of the strongest enhancements on a percent basis are for relatively low (2–6 spikes/sec) unenhanced spike rate responses, consistent with the Principle of Inverse Effectiveness.

### Establishing the domain of the power law

**Figure 4:**
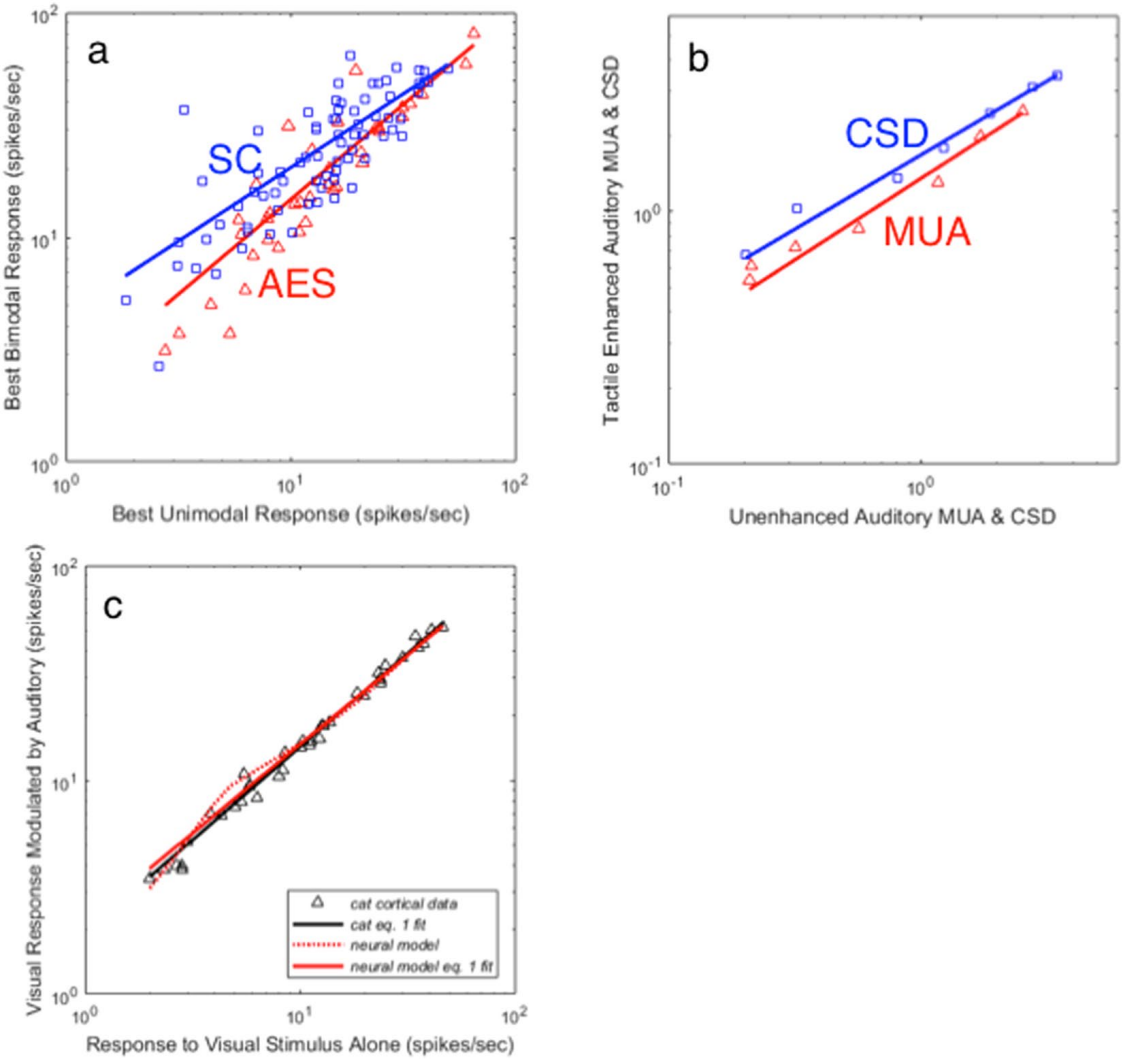
Power law fits to other kinds of data. (**a**) Bimodal cells (from cat superior colliculus (SC) and from cat cortical area AES^23^) are functions of two variables, so the single-variable fit of Eq. 1 to bimodal cell firing rates should be poorer than for the subthreshold neurons in Fig. 2, as indeed they are (see Table 1). (**b**) Power laws fits to two other electrophysiological measures – Multi Unit Activity and Current Source Density – have good r^2^ values but more variety in exponents than the Fig. 2 data. Units for CSD are mV/mm^2^; units for MUA are μV. Data shown are from the supergranular layer of primate auditory cortex (A1)^12^. See Table 1 for fits to other neural layers. (**c**) A neural model of spike rate amplification^24^ produces an amplification function (red dotted line) that is approximated by a power law (red solid line), but shows small systematic deviations at low firing rates. Black data points and black solid line are actual spike rate data (and power law fit) from Allman *et al*.^17^ for comparison (reproduced from the black data and fitted line shown in Fig. 2). The neural model (red dotted line) is superimposed on the neural data, not fit to it.

It is worth seeking excursions from the amplification function power law behaviors, both to establish the domain of Eq. 1 and because exceptions can be instructive. Figure 4 explores three cases.

Figure 4a shows power law fits to two sets of bimodal cells, one set from superior colliculus and one set from cortical area AES, both taken from the same study^23^. Since the amplified response produced by bimodal cells is (by definition) a function of two variables, a simple power law should not produce as good a fit to bimodal cell firing rates as it does to the firing rates of subthreshold neurons, and indeed it does not, especially for superior collicular (midbrain) neurons (r^2^ = 0.63). However, the fit is somewhat better for cortical AES bimodal cells (r^2^ = 0.83) and the resulting exponent (0.84) is within the range of the exponents found for subthreshold cells (Table 1). Similar results were found for other cortical bimodal cells in areas AES and PLLS (see Table 1). This is interesting because superior collicular bimodal and cortical subthreshold neurons are sometimes thought of as two points along a multisensory processing continuum^22^, and Meredith’s findings^23^ from AES (reanalyzed in Fig. 4a) argue that cortical bimodal neurons lie somewhere in the midst of that continuum.

Figure 4b explores electrophysiological data other than spike rates. The data shown are for current source densities and multiunit activity in macaque auditory cortex^12^. Current source densities (CSD) and multiunit activity (MUA) are thought to reflect massed synaptic and axonal activity respectively^12^. Figure 4b shows only the data from one neural layer, but both fits from each of three sampled cortical layers are shown in Table 1. Although the exponents of the power laws vary, the power law fits are quite good and consistent with a function of a single variable (a gated amplification). This speaks to a hypothesis raised by Laurienti *et al*.^7^, that measures of mass activity in cortex will likely reflect the actions of the more cortically numerous neurons that do not produce large amplifications (a group that would include subthreshold neurons).

Finally Fig. 4c looks at the predictions of a model of spike rate amplification in simulated synaptically-coupled subthreshold multisensory neurons^24^. The amplification function produced by the model is shown by a dotted red line. The amplification function is well approximated by a power law (solid red line; *n* = 0.83; r^2^ = 0.97), but a cursory examination shows small systematic deviations from the power law at low spike rates, suggesting a venue for additional modeling. Real data from the cat cortical neurons in Fig. 1b (black triangles)^17^ and the Eq. 1 fit to that data, are superimposed on the model for comparison only (e.g., the model is computed from first principles, instead of being fit to the data).

## Discussion

In summary, sensory enhancement for psychophysical data and for all electrophysiological data (except data from bimodal cells) is consistent with gated (triggered) power law amplification. Nothing about the second signal − other than its presence as an amplification trigger − is required to predict the amplification of the enhanced sensory response from the unenhanced response. The power law exponent varies from slightly compressive (0.9) to more compressive (0.6), consistent with the amplification of weak stimuli, relative to strong ones, found throughout the sensory literature. If the generality of these findings hold up, this may have other consequences. For example, S.S. Stevens and his associates spent much effort describing power law responses for single sensory responses; there is a rich database here covering many sensory variables^28^. Figure 1a shows a nice illustration of this – Stein’s lab^4^ finds a Stevens’ law for brightness of (quiet) lights, with an exponent of 0.6. They also found that the Stevens’ law for enhanced data has an exponent of 0.5. The generality of the results of this paper suggest that estimates for Stevens’ laws for multisensory amplifying conditions (in various senses), could be obtained by multiplying the exponents that Stevens obtained by about 0.85 (the average of the four psychophysical exponents from the amplification functions shown in Table 1).

Compressive nonlinearities like those usually found in Stevens’ law are common in electrophysiology and psychophysics and contribute to a larger dynamic range for sensory systems. The compressive exponents found here can be a second order effect arising from interactions of already compressive sensory systems. For example, in Fig. 4c, a binding-theory-based model of amplification employs mutual excitatory synaptic connections between two responding sensory systems, and has been used to model amplification in rattlesnakes and cats^24^. The mutual excitation results in amplification as each system enhances the other’s firing rates. The compression in this case arises partly from spike rate compression in the firing neurons and partly from a simulated synaptic depression, which tempers amplification at high firing rates more than low.

The similarity of the amplification functions for sensory integration and chromatic brightness (Fig. 2, Table 1) are intriguing for three reasons. First, it implies that the mechanisms involved may be quite generic and may be applicable to other psychophysical phenomena. Second, it provides a fascinating alternate explanation of the origins of chromatic brightness, a fundamental color percept. Third, it prompts consideration of the relationship between different kinds of neural information integration problems. This has a precedent in multisensory integration. For example, rattlesnake optic tectum integrates infrared information from their facial pits and visual information from their eyes and this is considered multisensory integration^29,30^, but if the rattlesnake’s infrared transduction had taken place in the retina, the integration of the infrared and visual spectral bands would almost certainly be considered an aspect of color vision.

Finally, it is surprising that so much perceptual and neural data can be accounted for as a gated amplification of an underlying sensory variable, because there is a rich literature on linear and nonlinear weighted cue combinations, and on strong- and weak-fusions of sensory information (see^31^ for recent reviews). The phenomena described here and the phenomena described in the cue combination literature seem to belong to different perceptual regimes and it would be interesting to understand whether the gulf between them is fixed and sharply delineated, or to explore the perceptual processing regime between them.

### Theoretical Methods

Data from ref.^17^ was obtained from the authors. Data from refs^10,32,33^ were taken from published tables. Data from refs^4,9,12,15,19,23^ were digitized from greatly magnified graphs using digital calipers (Lakatos provided better graphs than those available in ref.^12^ for this purpose). Datasets that had too many closely packed unresolvable data points were not employed unless the author made the data available. The data (red dotted line) shown in Fig. 4c (labeled neural model) was computed from a model^24^ that consists of two simulated Hodgkin-Huxley-like neurons with mutual excitatory synaptic coupling. The eight coupled differential equations that represent these spiking neurons and their synapses were integrated using a fourth-order Runge-Kutta algorithm in MATLAB.
